# Spontaneous Intestinal Tumorigenesis in *Apc* ^/Min+^ Mice Requires Altered T Cell Development with IL-17A

**DOI:** 10.1155/2015/860106

**Published:** 2015-06-03

**Authors:** Wook-Jin Chae, Alfred L. M. Bothwell

**Affiliations:** Department of Immunobiology, Yale University School of Medicine, New Haven, CT 06520, USA

## Abstract

The control of inflammatory diseases requires functional regulatory T cells (Tregs) with significant Gata-3 expression. Here we address the inhibitory role of Tregs on intestinal tumorigenesis in the *Apc* ^/Min+^ mouse model that resembles human familial adenomatous polyposis (FAP). *Apc* ^/Min+^ mice had a markedly increased frequency of Foxp3+ Tregs and yet decreased Gata-3 expression in the lamina propria. To address the role of heterozygous *Apc* gene mutation in Tregs, we generated *Foxp3-Cre*, *Apc* ^flox/+^ mice. Tregs from these mice effectively inhibited tumorigenesis comparable to wild type Tregs after adoptive transfer into *Apc* ^/Min+^ mice, demonstrating that the heterozygous *Apc* gene mutation in Tregs does not induce the loss of control over tumor microenvironment. Adoptive transfer of in vitro generated *Apc* ^/Min+^ iTregs (inducible Tregs) failed to inhibit intestinal tumorigenesis, suggesting that naïve CD4 T cells generated from *Apc* ^/Min+^ mice thymus were impaired. We also showed that adoptively transferred IL-17A-deficient *Apc* ^/Min+^ Tregs inhibited tumor growth, suggesting that IL-17A was critical to impair the tumor regression function of *Apc* ^/Min+^ Tregs. Taken together, our results suggest that both T cell development in a functional thymus and IL-17A control the ability of Treg to inhibit intestinal tumorigenesis in *Apc* ^/Min+^ mice.

## 1. Introduction

The intrinsic connection between inflammation and cancer promotion is well established [1]. The main role of regulatory T cells (Tregs) is to suppress inflammation [2]. The generation of peripheral Tregs is particularly important in the intestinal tract, and about 20–30% of Tregs in the intestine are adaptively induced Tregs (iTregs) [3, 4]. Tregs express a lineage specification marker Foxp3 and they use various types of suppression mechanisms including the secretion of immunosuppressive cytokines (e.g., IL-10 and TGF-*β*) and cell-cell contact [2, 5]. In addition to the expression of Foxp3, a very recent study demonstrated that another Th2 type CD4 T cell lineage marker Gata-3 is critical to maintain homeostasis and suppressive function of Tregs in suppressing intestinal inflammation* in vivo* [6, 7].

There is a high incidence of Tregs in tumor infiltrating lymphocyte (TIL) populations [8]. In most human carcinomas, these Tregs have been considered as an obstacle for effective antitumor immunity. A variety of localized and metastatic human carcinomas showed that the accumulation of Tregs in the tumor microenvironment is associated with unfavorable disease outcome [8]. This has been also demonstrated in experimental animal models of cancer [9–12]. In human colorectal cancer, however, a series of reports argued that the high incidence of tumor infiltrating Tregs in patient samples predicted improved survival or better disease outcome [13]. In line with this, a previous study showed that the adoptive transfer of splenic wild type Tregs effectively regressed intestinal tumors in *Apc* ^/Min+^ mice [14].

The *Apc* ^/Min+^ mouse model is well established to investigate the mechanisms of early stages of spontaneous intestinal tumorigenesis. It resembles human FAP (familial adenomatous polyposis), and many genetic/therapeutic approaches were tested in this model [15]. *Apc* ^/Min+^ mice and human FAP patients possess an inherited heterozygous germline mutation in the* Apc* gene that results in a truncated form of APC protein. The* Apc* gene undergoes further loss of heterozygosity (LOH) via the deletion of the wild type* Apc* allele and this results in activation of excessive Wnt signaling that drives polyp formation in the intestinal tract [16]. FAP patients develop colon polyps early in life and all affected individuals develop cancer. In contrast, sporadic colon cancer patients do not have a germline mutation in the* Apc* gene and generally develop disease much later in life. However, the role of Treg cells in the rapid onset of intestinal tumorigenesis *Apc* ^/Min+^ mice has not been addressed.

In the current study, we characterized *Apc* ^/Min+^ mice Tregs in the LP and investigated the role of the heterozygous Apc gene mutation in Tregs in a spontaneous intestinal tumorigenesis model. Tregs from IL-17A deficient *Apc* ^/Min+^ mice were used to assess the effect of the tumor environment and functional thymus.

## 2. Materials and Methods

### 2.1. Mice

*Apc* ^Min/+^ mice were purchased from the Jackson Laboratory and have been bred and maintained in our SPF mouse facility in conventional housing condition. All other mouse strains mentioned below were also housed in our SPF mouse facility in conventional housing condition in suspended stainless wire-mesh cages. Mouse health status was regularly monitored by Yale University Institutional Animal Care and no infections were reported in animals used in this study. The animals were kept under normal light/dark cycle. In all experiments, wild type (WT) littermates of *Apc* ^Min/+^ mice on the C57BL/6 background were used. IL-17A KO-*Apc* ^Min/+^ mice were generated previously in C57Bl/6 background [17]. Foxp3-IRES-RFP mice (FIR) were provided by Richard Flavell (Yale University) and bred with *Apc* ^Min/+^ mice and C57BL/6 mice to generate *Apc* ^Min/+^-Foxp3-IRES-RFP (*Apc* ^Min/+^-FIR) mice in C57Bl/6 background.* Foxp3-Cre* transgenic mice were generously provided by Alexander Rudensky (Sloan-Kettering Cancer Center) and maintained in our colonies for more than 6 generations in C57Bl/6 background. *Apc* ^flox/flox^ mice in C57Bl/6 background were obtained from the NCI and reported previously [18]. This strain was bred to* Foxp3-Cre* transgenic mice resulting in truncation of the Apc protein at amino acid 580 in Foxp3+ cells. All mouse protocols were approved by the Yale University Institutional Animal Care and Use Committee in accordance with the Association for Assessment and Accreditation of Laboratory Animal Care International. Genotyping conditions and primer sequences for mouse strains used in this study are as follows.

*Apc* ^Min/+^ mice: 94°C 4 min, (94°C 45 sec, 64°C 45 sec, −0.3°C 45 sec/cycle, 72°C 1 min) 35 cycles, 72°C 5 min.

Forward primer 5′-ttc cac ttt ggc ata agg c-3′, Reverse primer, 5′-ttc tga gaa aga cag aag tta-3′.

Foxp3-IRES-RFP mice: 94°C 3 min, (94°C 30 sec, 65°C 30 sec, −0.3°C 45 sec/cycle, 72°C 40 sec) 36 cycles, 72°C 5 min. Primer 1, 5′-caaaac caagaaaaggtgggc-3′; Primer 2: 5′-ggaatgctcgtcaagaagacagg-3′; Primer 3: 5′-cagtgctgttgctgtgtaagggtc-3′.

*Apc* ^flox/flox^ mice: 94°C 5 min, (94°C 30 sec, 58°C 30 sec, 72°C 45 sec) 32 cycles, 72°C 5 min. Forward primer 5′-gag aaa ccc tgt ctc gaa aaa a-3′, Reverse primer, 5′-agt gct gtt tct atg agt caa c-3′.

*Foxp3-Cre* transgenic mice: 94°C 3 min, (94°C 30 sec, 52°C 1 min, 72°C 1 min) 35 cycles, 72°C 2 min. Forward primer, 5′-agg atg tga ggg act acc tcc tgt a-3′, Reverse primer, 5′-tcc ttc act ctg att ctg gca att t-3′.

IL-17A KO mice: 94°C 2 min, (94°C 15 sec, 64°C 30 sec, 72°C 1 min) 40 cycles, 72°C 5 min 10 min. primer 1, 5′-actcttcatccacctcacacga-3′; primer 2, 5′-gccatgatatagacgttgtggc-3′; primer 3, 5′-cagcatcagagactagaaggga-3′. Primers 1 and 2 were used to detect wild type allele, and primers 1 and 3 were used to detect mutant allele.

### 2.2. Tumor Counts and Size

Animals were examined for mortality and clinical signs throughout the study. All mice surviving to the end of study were euthanized for determining tumor size and number. Following euthanasia, the entire small intestinal tract from each mouse was immediately removed and divided into 3 sections from the duodenum to the ileum. The intestinal sections were opened, and a dissecting microscope (magnification 20x) was used to identify tumors in each section. A calibrated reticle in an eyepiece of the dissecting scope was used to measure the diameter of each tumor at its maximum dimension. Lesions less than 0.5 mm in diameter were not enumerated. Large tumors (>2 mm) or small tumors (0.5–2 mm) were counted.

### 2.3. Adoptive Transfer Model and Treg Depletion

For the isolation of *Apc* ^/Min+^ Tregs and wild type Tregs, spleens from 6-7-week-old *Apc* ^Min/+^-Foxp3-IRES-RFP mice and their littermate controls (Foxp3-IRES-RFP) were prepared as single cell suspensions. After RBC (red blood cell) lysis, cells were stained with CD16/CD32 FcR (Fc Receptor) antibody (clone 2.4G2) to block nonspecific binding and then subsequently stained with anti-CD4 (clone RM4-5) antibody. RFP+ cells were sorted by FACSVantage and then transferred immediately to 3-month-old *Apc* ^Min/+^-Foxp3-IRES-RFP mice. RFP+ cells were subsequently sorted and then immediately transferred into 3-month-old *Apc* ^Min/+^ mice. For Apc mutant Tregs (Apc HET Tregs), splenic CD4+YFP+ cells were isolated from 6-week-old* Foxp3-Cre*; *Apc* ^flox/+^ mice; and* Foxp3-Cre* transgenic mice. CD4+CD45RBlowCD25-splenic Tregs were isolated from 6–8-week old IL-17A KO-*Apc* ^Min/+^ mice. For each transfer experiment, 4 × 10^5^ cells were injected per animal intravenously. Mice were analyzed on week 16. To perform cellular analysis, mice were sacrificed and LP (lamina propria) cells were prepared. For histological analysis, the terminal ileum from each mouse was dissected, fixed in 10% formalin, and washed in PBS solution. The specimen was paraffin-embedded, sectioned at 5 *μ*m, and stained with hematoxylin and eosin (H&E).

### 2.4. Antibodies and Reagents

Anti-mouse CD4 (clone RM4-5), anti-Helios (clone 22F6), anti-Foxp3 (clone FJK-16s), anti-ROR*γ*t (clone AFKJS-9), anti-GATA-3 (clone TWAJ), anti-Tbet (clone eBio4B10), anti-IL-17A (clone eBio 17B7), anti-IFN-*γ* (clone XMG1.2), CD45RB (clone C363.16A), Rag IgG2a isotype control (clone eBR2a), anti-CD3 (clone 145-2C11), and anti-CD28 (clone 37.51) were purchased from eBioscience. Human TGF-*β*1 was purchased from R&D systems. Diphtheria toxin and collagenase type IV were purchased from Sigma. DNase I was purchased from Roche.

### 2.5. Flow Cytometry and Cell Culture

For CD4 T cell differentiation, naïve CD4 T cells (CD4+CD62LhiCD44loFoxp3(RFP)-) were isolated from 6-7-week-old *Apc* ^Min/+^-Foxp3-IRES-RFP and littermate control mice. For inducible Treg transfer, splenic naïve CD4+ T cells from 6-week-old *Apc* ^Min/+^-Foxp3-IRES-RFP mice and their littermate controls (Foxp3-IRES-RFP) spleens were activated with anti-CD3 (2.5 *μ*g/mL), IL-2 (100 U/mL), and 1.5 ng/mL of TGF-*β* for 5 days. For LPMC (lamina propria mononuclear cell) preparation, 16-week-old *Apc* ^Min/+^ mice and Treg-transferred *Apc* ^Min/+^ mice were sacrificed, and the individual small intestines were processed as described with modification [19]. Briefly, small intestines were cut longitudinally and fecal contents were removed. Each small intestine was cut into 5 cm long pieces and extensively washed with PBS. Subsequently, small intestine pieces were incubated in shaking incubator with 5 mM EDTA-PBS solution. To digest these pieces, EDTA was removed and collagenase and DNase I solution was added in 5% FBS RPMI media for 30 min at 37°C. After Percoll gradient separation, cells were washed with ice-cold PBS twice, and lymphocytes were counted with trypan blue exclusion. Approximately 90% of cell viability was obtained. The yield of cells was presented in Supplementary Figure 1 in Supplementary Material available online at http://dx.doi.org/10.1155/2015/860106. For the stimulation of LPMC cells, PMA (100 ng/mL) and ionomycin (1 *μ*M) were used. Brefeldin A (1 *μ*g/mL) was added for the 4 h of culture in a total 6 hr of activation. After stimulation intracellular staining was performed as described in the manufacturer's protocol (eBioscience). A FACSCalibur (BD Biosciences) was used for flow cytometry and data were analyzed by FlowJo software (Treestar).

### 2.6. Real Time PCR

Cells were harvested and washed with PBS and then suspended in Trizol. cDNA was synthesized with the BDsprint cDNA synthesis kit (Clontech) and used with SYBR Green (Molecular Probes). Real-time PCR was performed using a Real time Bioanalyzer (BioRad). Primers were synthesized by the Keck Biotechnology Institute (Yale University). We calculated relative gene expression by the Δ*C*_*T*_ method [20]. The mRNA expression was normalized against HPRT. Primer sequences are available upon requests. PCR reaction was done using the following cycle sequence: 95°C 3 min, 95°C 30 s, 61°C 20 s, and 72°C 45 s for 40 cycles.

### 2.7. Statistical Significance

Statistically significant differences were determined by two-tailed unpaired Student's *t*-test (*p* < 0.05 was taken as significant) or one-way ANOVA analyses followed by Bonferroni's post hoc test with Graph Pad Prism software (GraphPad Software).

## 3. Results

### 3.1. Regulatory T Cells Are Selectively Expanded and Altered in the Lamina Propria of *Apc* ^/Min+^ Mice

**Figure 1:**
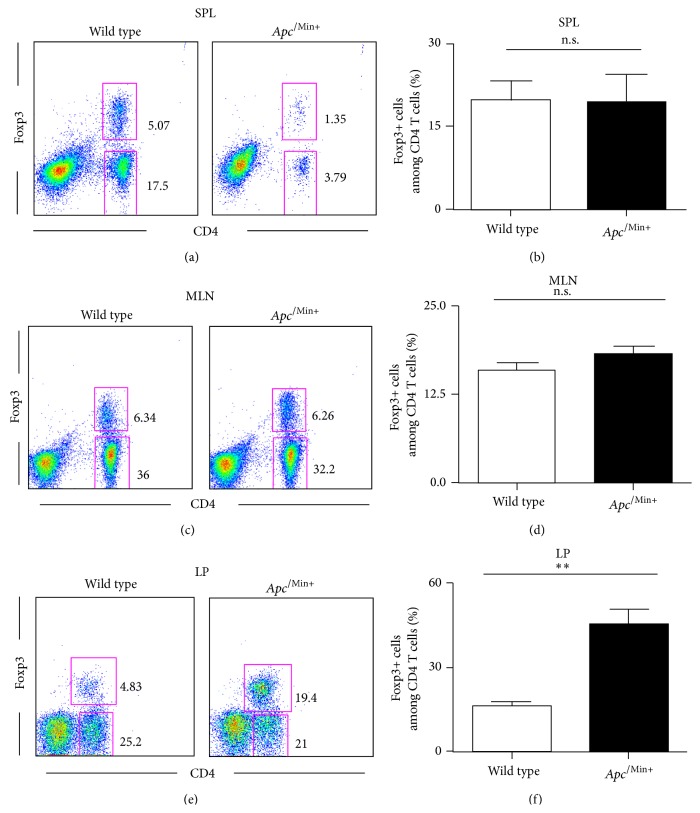
Regulatory T cells are selectively expanded and altered in the lamina propria of *Apc* ^/Min+^ mice. (a–d) Four-month-old *Apc* ^Min/+^ mice and their littermate controls (*n* = 3 for each group) were sacrificed. Single cell suspensions from spleen, mesenteric lymph nodes (MLN), and lamina propria (LP) were counted and analyzed by flow cytometry. The percentage of Foxp3+ cells among CD4 T cells was presented in the bar graph. A representative of three independent experiments is shown. Two-tailed *t*-test was performed. ^*∗∗∗*^ *p* < 0.0005; ^*∗∗*^ *p* < 0.005; ^*∗*^ *p* < 0.05; n.s.: not significant.

We first decided to characterize the Treg cell compartment in *Apc* ^/Min+^ mice. We examined the frequencies of Foxp3+ Treg among CD4 T cells in *Apc* ^/Min+^ mice and their littermate controls. In the spleen, there was no substantial enrichment of CD4+Foxp3+ cells in *Apc* ^/Min+^ mice at week 16 (Figure 1(a)). The frequencies of CD4 T cells were reduced in *Apc* ^/Min+^ mice although the total number of splenocytes was markedly increased (Figure 1(b)). This reduced frequency of CD4 T cells was previous reported and attributed to lymphodepletion [21]. In mesenteric lymph nodes (MLNs), CD4 T cell and Foxp3+ cell populations were largely unaltered (Figures 1(c) and 1(d)). Interestingly, we showed that there is an approximately 2.5-fold increase in the frequency of Foxp3+ Treg in the LP of *Apc* ^/Min+^ versus wild type C57BL/6 mice (Figure 1(e)), suggesting that *Apc* ^/Min+^ Treg expansion is specific to the LP. We also showed that *Apc* ^/Min+^ mice had about 2.5 times more cells in the LP (Figure 1(f) and Supplementary Figure 1). Taken together, our results show that the accumulation of Foxp3+ Treg is specifically found in the LP where intestinal tumorigenesis occurs.

### 3.2. Th17 Cells and Altered Foxp3+ Tregs Cells Are Both Increased in the Lamina Propria of *Apc* ^/Min+^ Mice

**Figure 2:**
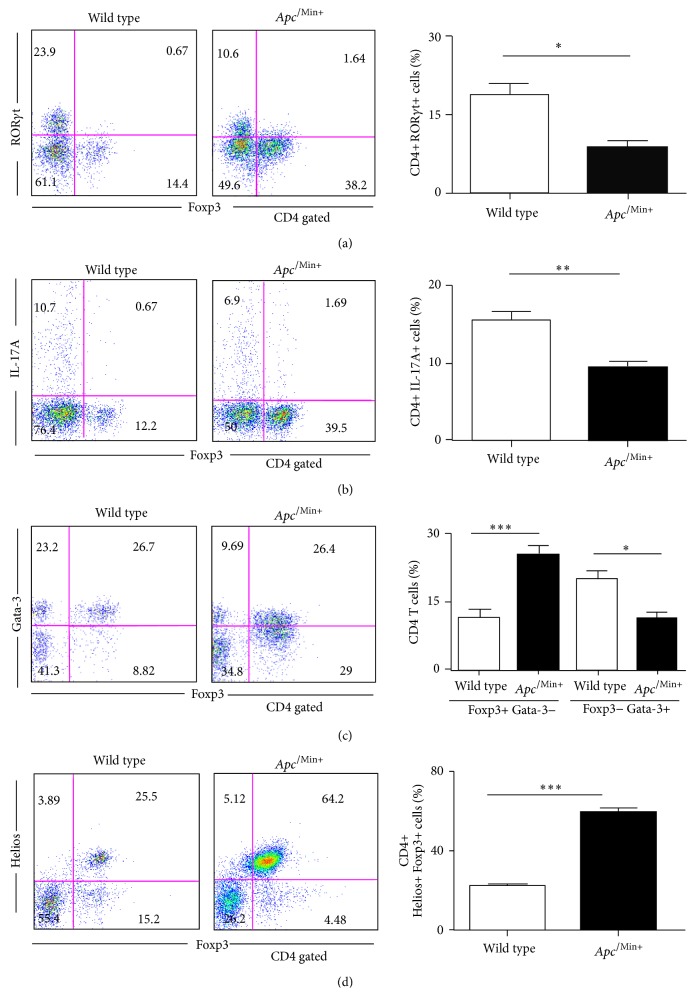
Th17 cells and altered Foxp3+ Tregs cells are both increased in the lamina propria of *Apc* ^/Min+^ mice. (a–d) Four-month-old *Apc* ^Min/+^ mice and their littermate controls (*n* = 3 for each group) were sacrificed. Single cell suspensions of LP cells were analyzed by flow cytometry. LP cells were stimulated for 6 hrs to detect IL-17A. A representative of three independent experiments is shown. Two-tailed *t*-test was performed. ^*∗∗∗*^ *p* < 0.0005; ^*∗∗*^ *p* < 0.005; ^*∗*^ *p* < 0.05; n.s.: not significant.

The tumor microenvironment alters the effector phenotype of CD4 T cells and the balance with regulatory T cells [8, 22]. We decided to characterize Th17 cells and Treg in the LP of *Apc* ^/Min+^ mice. First, we measured the expression of the Th17 lineage transcription factor ROR*γ*t in the LP CD4 T cells from wild type and *Apc* ^/Min+^ mice. As Th17 cells are important to initiate intestinal tumorigenesis, we hypothesized that the levels of ROR*γ*t+ and IL-17A+ CD4 T cells in the LP of *Apc* ^/Min+^ mice could be substantially elevated. Unexpectedly, the frequency of ROR*γ*t+ CD4 T cells was decreased in the LP compared to control mice while that of Foxp3+ cells was increased (Figure 2(a)). We also examined the expression of IL-17A in the LP as markers for expression for Th17 cells (Figure 2(b)). Importantly, the highly expanded *Apc* ^/Min+^ Foxp3+ Treg in the LP did not coexpress substantial levels of ROR*γ*t or IL-17A (Figures 2(a) and 2(b)). Although the frequency of IL-17A+ CD4 T cells was reduced by 30%, the 2.5-fold increase of total LP cells resulted in only moderate increased of IL-17A+ CD4 T cells (Figure 2(b) and Supplementary Figure 1). Thus, the tumor microenvironment in *Apc* ^/Min+^ mice have markedly increased numbers of Foxp3+ Tregs but only moderately increased IL-17A+ CD4 T cells in the LP, suggesting that *Apc* ^/Min+^ Foxp3+ Tregs are altered in the LP.

We further examined possible alterations in *Apc* ^/Min+^ Foxp3+ Treg. It has been reported that Gata-3 is a critical transcription factor needed to maintain Treg function* in vivo* [6]. In addition, it has been shown that Gata-3 is required for IL-10 expression and IL-10 is critical for inhibiting intestinal polyposis [14, 23]. As endogenous *Apc* ^/Min+^ Treg in the LP clearly failed to regress tumors, we tested whether these expanded Treg stably express Gata-3. Interestingly, the percentage of the Gata-3-Foxp3+ *Apc* ^/Min+^ Treg population markedly increased in the LP (8.82% versus 29%) (Figure 2(c)). However, the absolute number of Foxp3+Gata-3+ Tregs was also increased in *Apc* ^/Min+^ mice. This suggests that the expansion of Gata-3-*Apc* ^/Min+^ Treg was more favored by the tumor microenvironment, giving more nonfunctional Treg. It has been shown that Helios, a member of the Ikaros family, is expressed in more mature and proliferating CD4 T cells and Treg as well as in thymic-derived Tregs [24–26]. We also showed that the frequency of Helios+ Foxp3+ Treg was markedly increased in *Apc* ^/Min+^ mice while Helios-Foxp3+ Treg were very low (Figure 2(d)). Interestingly, Helios+ cells were not increased in the CD4 effector population, suggesting that the tumor microenvironment favors the activation and proliferation of *Apc* ^/Min+^ Treg. Taken together, our results show that *Apc* ^/Min+^ Foxp3+ Tregs undergo robust expansion in the intestinal tumor microenvironment and these Treg have altered Gata-3 expression that is important for Treg function* in vivo*.

### 3.3. The Heterozygous* Apc* Gene Mutation in Treg Alone Does Not Impair Treg-Mediated Control of Intestinal Tumorigenesis

Next we questioned whether the heterozygous mutation of Apc gene in Treg is responsible for their dysfunction in intestinal tumorigenesis. It has been known that *Apc* ^/Min+^ mice display thymic atrophy and altered bone marrow subpopulations. Transplantation of wild type bone marrow into *Apc* ^/Min+^ mice does not correct these phenotypes; thus the developmental environment of immune cells is altered [21]. It has been demonstrated that adoptive transfer of wild type Treg could effectively inhibit intestinal tumorigenesis [14].

As the heterozygous mutation of the* Apc* gene does occur in all cell types in *Apc* ^/Min+^ mice, we decided to generate a Treg-specific heterozygous* Apc* gene deletion using* Foxp3-Cre*; *Apc* ^flox/+^ (or* Apc* HET) mice. This allowed us to assess the role of Treg-specific* Apc* gene mutation. Tregs from this strain of mice express a truncated form of Apc protein having 580 amino acids (*Apc* HET Treg). Unlike the complete deletion of the Apc gene in Foxp3-specific manner, lymphocytes in* Apc* HET mice develop in a mouse with a wild type hematopoietic system (Supplementary Figure 2). We isolated splenic* Apc* HET Tregs based on YFP expression and transferred them into 3 month-old *Apc* ^/Min+^ mice.

**Figure 3:**
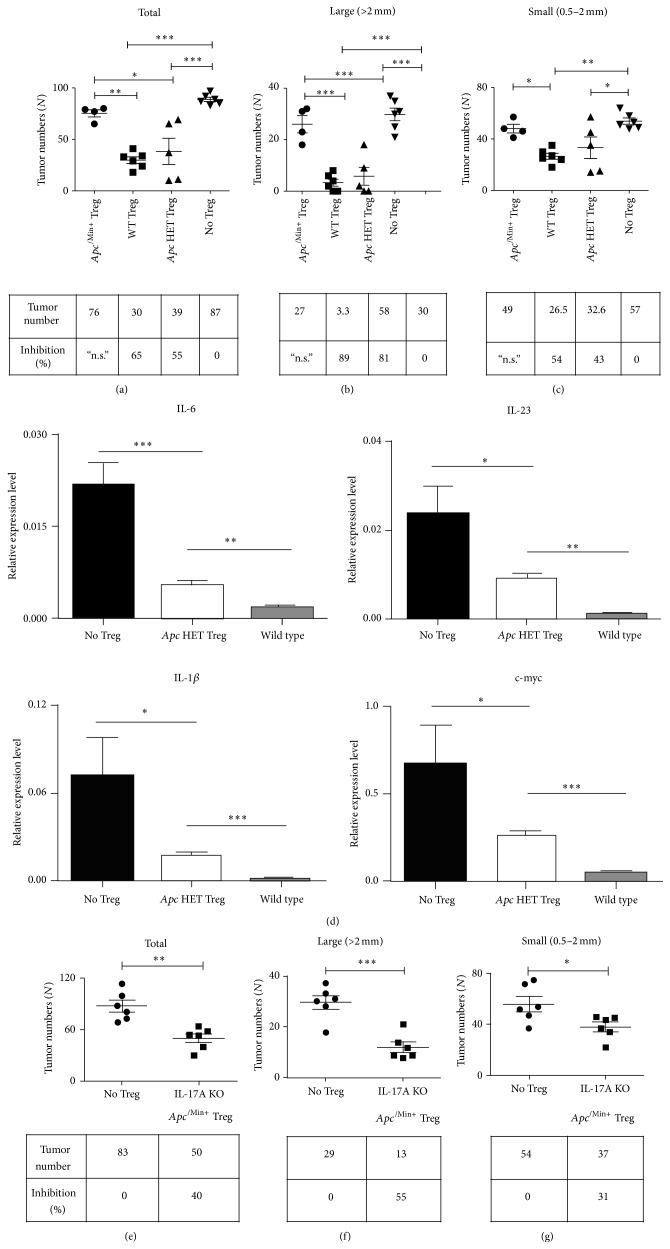
The heterozygous* Apc* gene mutation in Treg alone does not impair Treg-mediated tumor regression. (a–c) 4 × 10^5^ CD4+Foxp3+ cells were isolated from 6-7-week-old *Apc* ^Min/+^-FIR mice, their wild type littermate controls (FIR mice), and* Foxp3-Cre*; *Apc* ^flox/+^ mice by cell sorting and then transferred to 3-month-old *Apc* ^Min/+^ mice. Tumor numbers were measured 4 weeks after adoptive transfer. Large tumors (>2.0 mm) and small tumors (0.5–2.0 mm) were counted. (d) Tumors (>2.0 mm) from each group of mice were excised and analyzed by real time PCR. Each group had more than 5 polyps analyzed. mRNA expression was normalized against HPRT for their relative expression level (HPRT = 1). (e–g) 4 × 10^5^ Tregs from 6-7-week-old IL-17A deficient (KO)-*Apc* ^Min/+^ mice were transferred to 3-month-old *Apc* ^Min/+^ mice (*n* = 6 per group). Tumor numbers were measured 4 weeks after adoptive transfer. Data are shown as mean ± SEM. For (a–c), one-way ANOVA analysis with Bonferroni's post hoc test was used. For other analyses, two-tailed *t*-tests were performed for all groups. ^*∗∗∗*^ *p* < 0.0005; ^*∗∗*^ *p* < 0.005; ^*∗*^ *p* < 0.05; n.s.: not significant.

**Figure 4:**
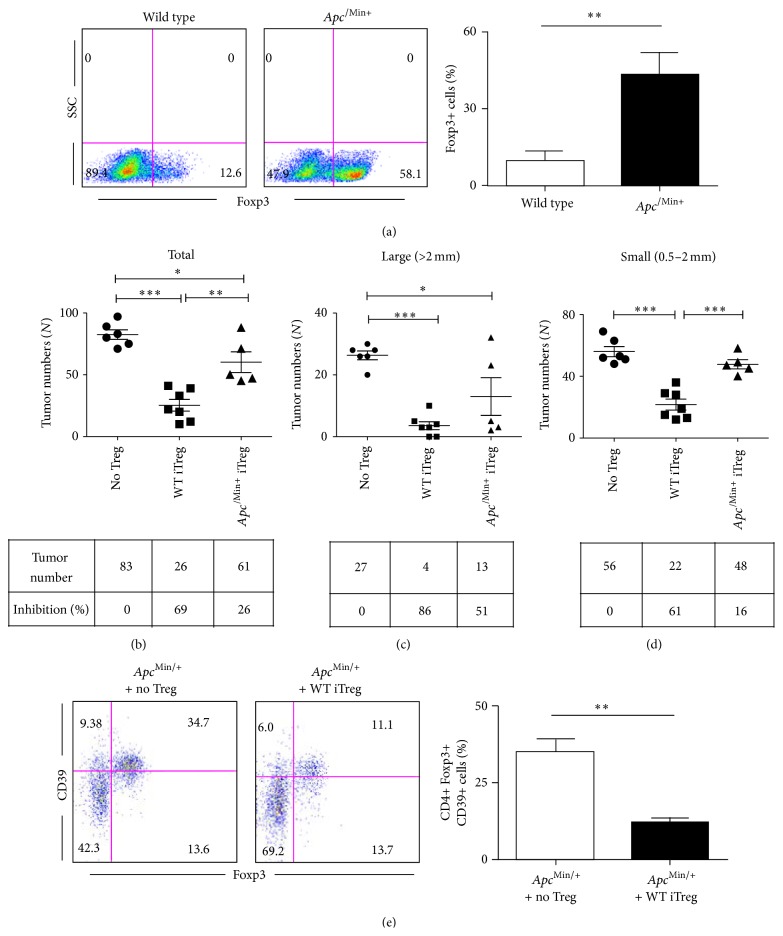
Wild type inducible Tregs can regress intestinal tumors in *Apc* ^Min/+^ mice. (a) Splenic naïve CD4 T cells from 6-7-week-old *Apc* ^Min/+^-FIR mice and their wild type littermate controls were isolated and differentiated into inducible Tregs (iTregs) for 120 hrs. A representative of three independent experiments is shown. ^*∗∗*^ *p* < 0.005. (b–d) Splenic naïve CD4 T cells from 6-7-week-old *Apc* ^Min/+^-FIR mice and their wild type littermate controls were isolated and differentiated into inducible Tregs (iTregs) and sorted. 4 × 10^5^ iTregs were transferred to 4 × 10^5^ (*n* = 7 for WT iTreg, *n* = 6 for *Apc* ^Min/+^ iTreg). Tumor numbers were measured 4 weeks after adoptive transfer. Data are shown as mean ± SEM. For (b–d), one-way ANOVA analysis with Bonferroni's post hoc test was used. For other analyses, two-tailed *t*-test was performed for all groups. ^*∗∗∗*^ *p* < 0.0005; ^*∗∗*^ *p* < 0.005; ^*∗*^ *p* < 0.05; n.s.: not significant. (e) LP cells from *Apc* ^Min/+^ mice that received WT iTregs were compared with *Apc* ^Min/+^ mice (*n* = 3) and analyzed by flow cytometry.

Interestingly,* Apc* HET Tregs showed a similar level of inhibition of total tumor numbers as wild type splenic Tregs (Figure 3(a)). Wild type Foxp3+ splenic Tregs potently decreased both large (Figure 3(b), 89%) and small intestinal tumors (Figure 3(c), 54%). The degree of inhibition by these* Apc* HET Tregs was more evident in large tumors (Figure 4(b) 81%) than small tumors (Figure 3(c) 46%). Further analysis of excised intestinal polyps showed that the adoptive transfer of* Apc* HET Tregs reduced the levels of proinflammatory mediators IL-6, IL-1*β*, and IL-23 in the tumor microenvironment compared with wild type intestinal tissue (Figure 3(d)) but the expression level of TNF was not decreased (Supplementary Figure 3). The transfer of* Apc* HET Tregs decreased the expression of c-myc in polyps, indicating that the aberrant proliferation in the intestinal tract has been inhibited (Figure 3(d)). Endogenous *Apc* ^/Min+^ Tregs were isolated from 6-7 week-old *Apc* ^/Min+^ mice. Importantly, *Apc* ^/Min+^ mice showed no readily visible tumors at this age in our colonies. Isolated *Apc* ^/Min+^ Tregs were adoptively transferred to 3-month-old *Apc* ^/Min+^ mice to compare their inhibitory capacity with* Apc* HET Treg. Interestingly, endogenous *Apc* ^/Min+^ Tregs were defective in their ability to decrease tumor numbers (Figures 3(a), 3(b), and 3(c)). This suggests that the direct effect of the heterozygous* Apc* gene mutation in Treg is minimal when* Apc* HET Treg cells develop in* a wild type* thymic environment.

Next, we questioned whether the exposure to tumor burden is important for *Apc* ^/Min+^ Tregs for their tumor regressor function. Previously, it has been demonstrated that IL-17A is a potent proinflammatory cytokine that drives intestinal tumorigenesis in *Apc* ^/Min+^ mice [17, 27, 28]. We previously showed that ablation of IL-17A in *Apc* ^/Min+^ mice achieved approximately 90% inhibition of polyposis even at 20 weeks, suggesting that the effect of IL-17A deficiency is long-lasting to inhibit the formation of a tumor microenvironment [17]. We questioned whether Treg with the *Apc* ^/Min+^ mutation from a mouse with a greatly reduced number of intestinal tumors could regress tumors. To this end, we isolated Treg from IL-17A deficient-*Apc* ^/Min+^ mice and transferred them into 3-month-old *Apc* ^/Min+^ mice. IL-17A deficient-*Apc* ^/Min+^ mice have very few tumors by 6-7 weeks, and thus Treg from this strain have not yet encountered a mature tumor microenvironment.

We observed that Tregs from IL-17A deficient *Apc* ^/Min+^ mice reduced tumors by 40% (Figure 3(e)). Large tumors were more effectively inhibited (55%) than small tumors (31%) (Figures 3(f) and 3(g)). These data suggest that the exposure to tumor burden is critical to impair *Apc* ^/Min+^ Tregs in the LP. Taken together, our results suggest that thymic T cell development and the exposure of *Apc* ^/Min+^ Tregs to the tumor microenvironment are critical to impair their capability for inhibiting intestinal tumorigenesis.

### 3.4. Wild Type Inducible Treg Can Decrease Intestinal Tumor Multiplicity

It has been also reported that the maintenance of adaptive Treg in the intestine is important to maintain homeostasis [29, 30]. We tested whether *Apc* ^/Min+^ naïve CD4 T cells are altered in their ability to generate inducible Tregs (iTreg). Interestingly, splenic *Apc* ^/Min+^ naïve CD4 T cells were more readily induced to become iTregs than their wild type counterparts (Figure 4(a)). There is approximately a 4-fold increase in Foxp3+ cells in the presence of TGF-*β* (Figure 4(a)). This suggests the possibility that Treg could be induced more readily in the tumor microenvironment. To test whether increased iTreg populations might correct intestinal tumorigenesis, we adoptively transferred* in vitro* generated wild type iTreg and *Apc* ^/Min+^ iTreg. Notably, wild type iTreg effectively reduced intestinal tumor numbers (69%, Figure 4(b)) while *Apc* ^/Min+^ iTregs showed only a partial inhibitory effect (26%). Wild type iTregs reduced both large tumors (86%, Figure 4(c)) as well as small tumors (61%, Figure 4(d)). This poor inhibitory capability of *Apc* ^/Min+^ iTreg was mainly observed in small tumors (16%, Figure 4(c)) rather than large tumors (51% reduction, Figure 4(d)). The limited capacity of *Apc* ^/Min+^ iTregs in terms of inhibiting tumor numbers suggests that these Tregs have been altered by the intestinal tumor microenvironment. This is also consistent with our results above that the impaired T cell development of *Apc* ^/Min+^ Treg affected their capacity to limit tumor multiplicity and sizes of tumors (Figures 4(a)–4(c)). Hence, both splenic *Apc* ^/Min+^ nTreg and iTreg were functionally impaired in *Apc* ^/Min+^ mice. The expansion of Treg in the LP was markedly inhibited by wild type iTreg transfer, suggesting that wild type iTreg inhibited the formation of tumor microenvironment that induces Treg cell expansion (Figure 4(e)). Our results suggest that *Apc* ^/Min+^ naïve T cells have an altered capacity for generating iTreg and are functionally impaired by the intestinal tumor microenvironment.

## 4. Discussion

In this study we demonstrated the importance of T cell development and tumor microenvironment in inhibiting intestinal tumor multiplicity by Tregs in *Apc* ^/Min+^ mice. Since the role of regulatory T cells is to control inflammation, yet they failed to inhibit tumor numbers, the accumulation of endogenous *Apc* ^/Min+^ Tregs in the LP raised a question on their role in the intestinal tumorigenesis. The marked reduction in frequency of Gata-3+ Treg cells as well as the increase of Gata-3-Tregs suggests that tumor microenvironment favored the expansion of these altered *Apc* ^/Min+^ Tregs.

It should be noted that the Treg-specific heterozygous Apc mutation did not alter the function of Tregs to limit in both large and small sizes of tumors markedly, suggesting the critical role of thymic development in Treg-mediated control of tumor multiplicity in *Apc* ^/Min+^ mice. Unlike *Apc* ^/Min+^ mice,* Apc* HET mice do not have a germline mutation in the* Apc* gene. APC protein is a large protein and has multiple *β*-catenin binding sites and thus it might be possible that different length of mutant APC protein between* Apc* HET Tregs and *Apc* ^/Min+^ Tregs (580 and 850 amino acids, resp.) might have resulted in differential results in our study. However, recently, it has been shown that canonical Wnt signaling negatively regulates suppressor function in colitis model using *Apc* ^/Min+^ Tregs [31]. Since shorter form of APC protein would result in enhanced Wnt signaling in Tregs with fewer *β*-catenin binding sites,* Apc* HET Tregs would have more severe loss of suppressor function compared to *Apc* ^/Min+^ Tregs. Therefore, it is likely that *Apc* ^/Min+^ Tregs is primarily impaired by dysfunctional thymic development in *Apc* ^/Min+^ mice. More future studies regarding the regulatory function of APC protein in Tregs are warranted. We previously showed that the lack of IL-17A in *Apc* ^/Min+^ mice completely restored thymic as well as splenic cellularity while *Apc* ^/Min+^ mice underwent thymic atrophy and splenomegaly [17]. As *Apc* ^/Min+^ iTreg that were generated from naïve CD4 T cells that did not encounter tumor microenvironment, it also suggests that both nTregs and iTregs in *Apc* ^/Min+^ mice are impaired in tumor inhibition function in an altered thymic development. Of note, *Apc* ^/Min+^ iTregs lost most of their inhibitory effect on tumor multiplicity yet they still retained a sizeable effect on inhibition of large tumors, suggesting that the defect in *Apc* ^/Min+^ iTregs is mainly in controlling tumor induction while their inhibitory capacity to control tumor growth in tumor microenvironment is conserved similar to IL-17A KO *Apc* ^/Min+^ Tregs. The adoptive transfer of Treg from IL-17A-deficient *Apc* ^/Min+^ mice and *Apc* ^/Min+^ iTreg also suggests that the reduced tumor burden and normal thymic development of T cells are important to control intestinal tumor multiplicity. Since the decrease of small tumor numbers (31%) was less potent than that of large tumors (55%), it could be noted that the role of IL-17A from *Apc* ^/Min+^ Tregs is more important for large tumor formation in IL-17A-mediated tumor microenvironment. Thus, our results argue that the control of tumor multiplicity by Tregs requires both the absence of IL-17A and a functional thymus.

While exogenous agents such as enterotoxigenic bacteria can induce and expand Th17 cells in the LP [27], our results suggest that the marked expansion of Th17 cells may not be required for spontaneous intestinal tumorigenesis but a moderate increase of Th17 cells is sufficient to induce tumorigenesis. It is possible that the biological potency of IL-17A is more important than Th17 cell expansion as IL-17A-deficient *Apc* ^/Min+^ mice showed marked reduction of intestinal tumorigenesis [17]. Previous reports have suggested that a sizable fraction of Treg may coexpress Foxp3 and IL-17 [32]. However, our direct analysis of cells from the LP in *Apc* ^/Min+^ mice did not find such a high percentage of IL-17A+Foxp3+ CD4 T cells. This is may be due to differences in the particular mouse housing facility, the mouse strain or the intestinal microenvironment, since IL-17 production is variable depending on gut microflora [33].

A series of recent findings in colon cancer implied that the role of accumulated Treg may be unique in that they perform a more inhibitory role on cancer development [10, 12, 13]. On the other hand, other studies reported controversial results regarding the antitumorigenic role of Treg in sporadic colorectal cancer patients [34, 35]. The heterogeneous nature of the human intestinal tumor microenvironment is likely to contribute to this controversy for the role of Treg. Therefore, the role of Treg may primarily depend on the tumor microenvironment in sporadic colon cancer, while in FAP patients there is also an altered T cell development issue that impairs the function of Treg to regress intestinal tumors.

As both FAP patients and *Apc* ^Min^ mice show typically a high incidence of rapidly developing tumors, our study is important to provide a potential clinical strategy for FAP in patients, particularly in the early stages of intestinal tumorigenesis where the role of Treg could be antitumorigenic. Our positive findings using wild type iTreg may also be of use clinically since the number of natural Tregs in human blood is low. Taken together, our results offer novel insight into the mechanism of early and rapid onset of FAP by implicating a lack of functional Treg cells as a major factor.

## 5. Conclusion

Our work suggests that thymic T cell development may be an important factor to consider in rapid intestinal tumorigenesis in *Apc* ^/Min+^ mice driven by IL-17A that resemble FAP patients who bear the germline* Apc* gene mutation, unlike from sporadic colorectal cancer (CRC).

## Supplementary Material

Supplementary Figure 1: The yield of LPMC in 16 week-old wildtype and Apc^Min/+^ mice (n=3) LPMCs were isolated in materials and methods. Two-tailed t-test was performed. ∗∗∗, p< 0.0005.
Supplementary Figure 2: The homozygous deletion of the Apc gene in Tregs results in thymic atrophy and lymph adenopathy. Spleens, lymph nodes and thymus from 4 week-old Foxp3-Cre;Apc^flox/flox^ mice and Foxp3-Cre;Apc^flox/+^ mice were presented. Total number of cells in peripheral lymph nodes (pLNs) are shown (n=3). Two-tailed t-test was performed. ∗∗∗, p < 0.0005.
Supplementary Figure 3: Apc HET Treg transfer does not reduce the expression of TNF-α expression in polyps. Tumors (> 2.0 mm) were excised from each mouse and TNF-α expression was analyzed by real time PCR. Each group had minimum number of polyps as n=5. mRNA expression was normalized against HPRT for their relative expression level (HPRT=1). Two-tailed t-test was performed. n.s., not significant.
