# Supplementary figures and images for: Spontaneous Intestinal Tumorigenesis in Apc /Min+ Mice Requires Altered T Cell Development with IL-17A
Source: J Immunol Res. 2015 Jun 3;2015:860106. doi: 10.1155/2015/860106 (PMC4469837; doi:10.1155/2015/860106)

## Supplementary Figure 1

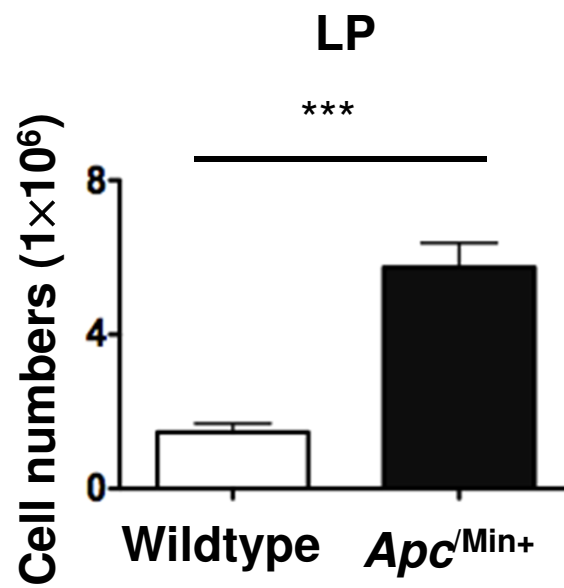

## Supplementary Figure 2

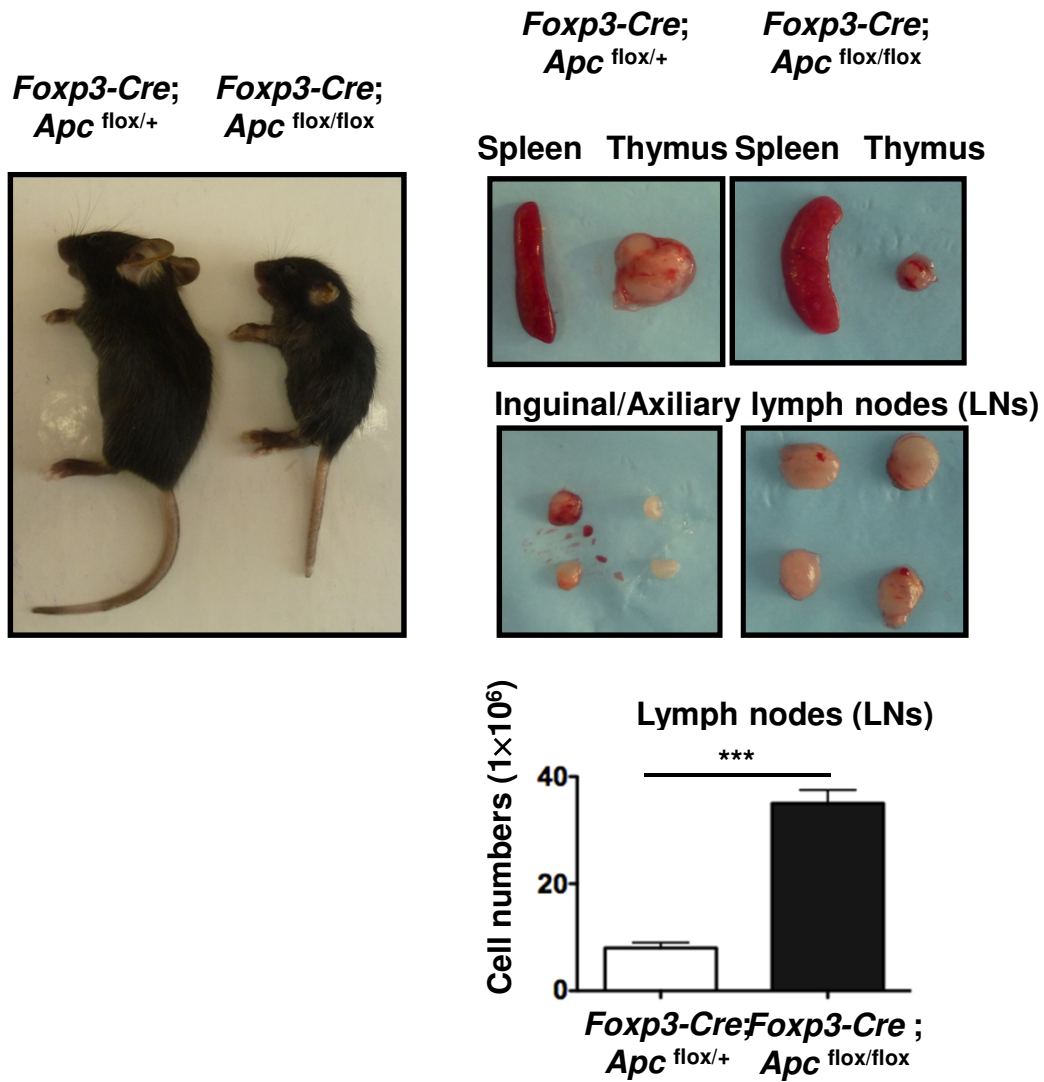

## Supplementary Figure 3

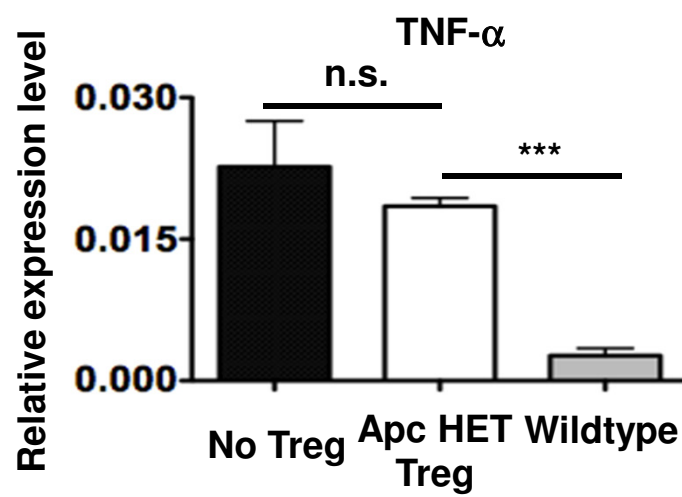

Supplement: Supplementary file 1 — Supplementary Figure 1: The yield of LPMC in 16 week-old wildtype and ApcMin/+ mice (n=3) LPMCs were isolated in materials and methods. Two-tailed t-test was performed. ∗∗∗, p< 0.0005. Supplementary Figure 2: The homozygous deletion of the Apc gene in Tregs results in thymic atrophy and lymph adenopathy. Spleens, lymph nodes and thymus from 4 week-old Foxp3-Cre;Apcflox/flox mice and Foxp3-Cre;Apcflox/+ mice were presented. Total number of cells in peripheral lymph nodes (pLNs) are shown (n=3). Two-tailed t-test was performed. ∗∗∗, p < 0.0005. Supplementary Figure 3: Apc HET Treg transfer does not reduce the expression of TNF-α expression in polyps. Tumors (> 2.0 mm) were excised from each mouse and TNF-α expression was analyzed by real time PCR. Each group had minimum number of polyps as n=5. mRNA expression was normalized against HPRT for their relative expression level (HPRT=1). Two-tailed t-test was performed. n.s., not significant. [file 860106.f1.pdf]
